# Supplementary material for: Integrated Analysis of Transcriptome, microRNAs, and Chromatin Accessibility Revealed Potential Early B-Cell Factor1-Regulated Transcriptional Networks during the Early Development of Fetal Brown Adipose Tissues in Rabbits
Source: Cells. 2022 Aug 28;11(17):2675. doi: 10.3390/cells11172675 (PMC9454897; doi:10.3390/cells11172675)
Supplement: Supplementary file 1 [file cells-11-02675-s001.zip › cells-1832136-supplementary/FigureS2-Integrated analysis of chromatin accessibility and gene expression during the early development of FBAT in rabbits.pdf]

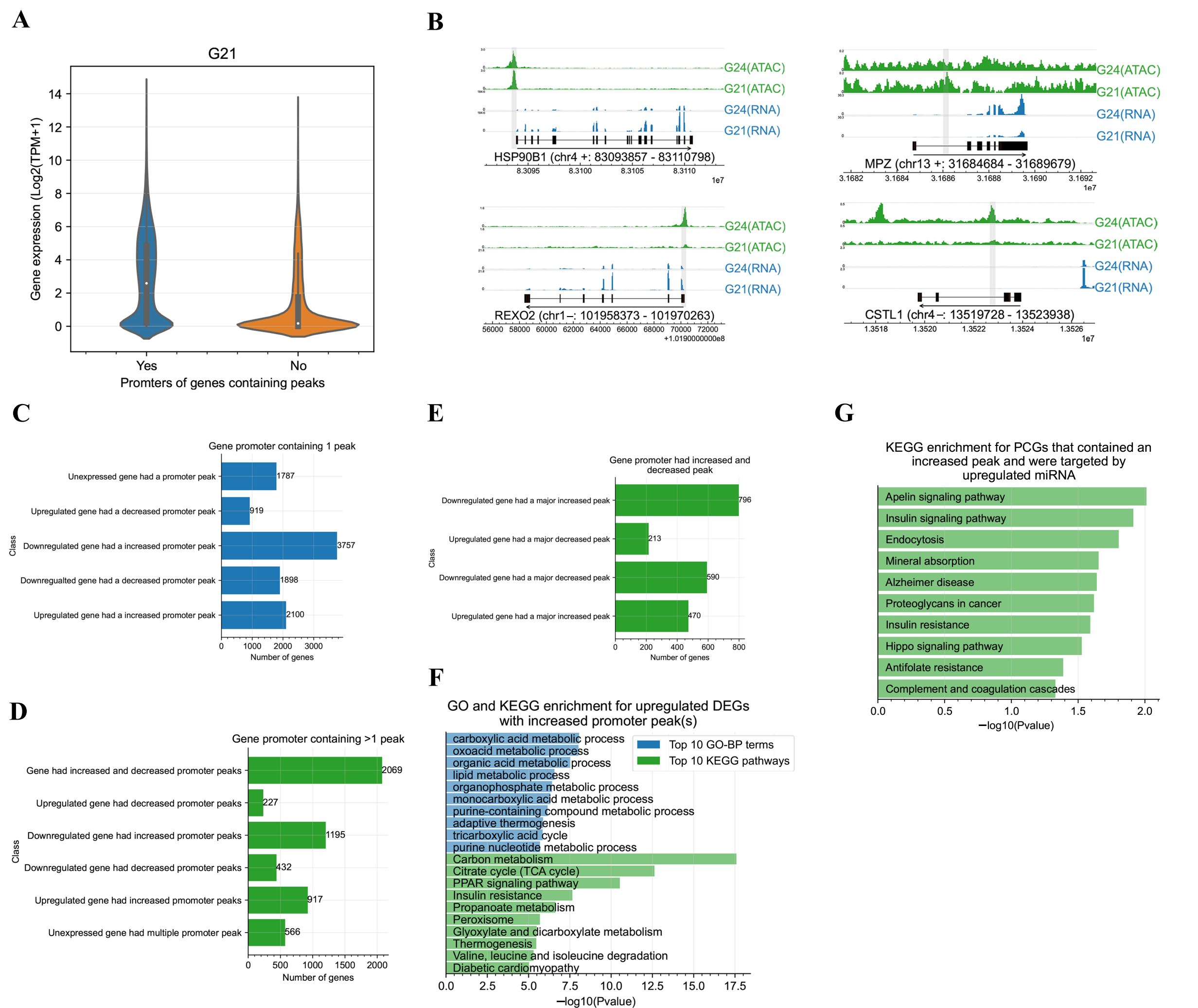
A
G21
Gene expression (Log2(TPM+1))
Yes
No
Promters of genes containing peaks
B
G24(ATAC)
G21(ATAC)
G24(RNA)
G21(RNA)
HSP90B1 (chr4 +: 83093857 - 83110798)
MPZ (chr13 +: 31684684 - 31689679)
REXO2 (chr1 –: 101958373 - 101970263)
CSTL1 (chr4 –: 13519728 - 13523938)
C
Gene promoter containing 1 peak
Unexpressed gene had a promoter peak 1787
Upregulated gene had a decreased promoter peak 919
Downregulated gene had a increased promoter peak 3757
Downregualted gene had a decreased promoter peak 1898
Upregulated gene had a increased promoter peak 2100
Class
Number of genes
D
Gene promoter containing >1 peak
Gene had increased and decreased promoter peaks 2069
Upregulated gene had decreased promoter peaks 227
Downregulated gene had increased promoter peaks 1195
Downregulated gene had decreased promoter peaks 432
Upregulated gene had increased pmomoter peaks 917
Unexpressed gene had multiple promoter peak 566
Class
Number of genes
E
Gene promoter had increased and decreased peak
Downregulated gene had a major increased peak 796
Upregulated gene had a major decreased peak 213
Downregulated gene had a major decreased peak 590
Upregulated gene had a major increased peak 470
Class
Number of genes
F
GO and KEGG enrichment for upregulated DEGs with increased promoter peak(s)
Top 10 GO-BP terms
Top 10 KEGG pathways
carboxylic acid metabolic process
oxoacid metabolic process
organic acid metabolic process
lipid metabolic process
organophosphate metabolic process
monocarboxylic acid metabolic process
purine-containing compound metabolic process
adaptive thermogenesis
tricarboxylic acid cycle
purine nucleotide metabolic process
Carbon metabolism
Citrate cycle (TCA cycle)
PPAR signaling pathway
Insulin resistance
Propanoate metabolism
Peroxisome
Glyoxylate and dicarboxylate metabolism
Thermogenesis
Valine, leucine and isoleucine degradation
Diabetic cardiomyopathy
−log10(Pvalue)
G
KEGG enrichment for PCGs that contained an increased peak and were targeted by upregulated miRNA
Apelin signaling pathway
Insulin signaling pathway
Endocytosis
Mineral absorption
Alzheimer disease
Proteoglycans in cancer
Insulin resistance
Hippo signaling pathway
Antifolate resistance
Complement and coagulation cascades
−log10(Pvalue)
