# Supplementary material for: Integrated Analysis of Transcriptome, microRNAs, and Chromatin Accessibility Revealed Potential Early B-Cell Factor1-Regulated Transcriptional Networks during the Early Development of Fetal Brown Adipose Tissues in Rabbits
Source: Cells. 2022 Aug 28;11(17):2675. doi: 10.3390/cells11172675 (PMC9454897; doi:10.3390/cells11172675)
Supplement: Supplementary file 1 [file cells-11-02675-s001.zip › cells-1832136-supplementary/Table S1.pdf]

Table S1 Primers of genes used in RT-qPCR

Gene ID	Ensembl ID	Primer sequences (forward/reverse)	Product length (bp)
CIDEA	ENSOCUG00000011380	TAGGGGACAACACGCACTTC/CTCTGGCGATTCCCGATCTC	105
UCP1	ENSOCUG00000002297	TGGGCACACTCGCATATTGT/TCGCTGGATTTTGCACAACG	96
PPARGC1A	ENSOCUG00000014668	AAAAGCTTGACTGGCGTCAC/ACTGCACCACTTGAGTCCAC	202
ND1	ENSOCUG00000029086	ACCCTAGCAGAAACCAACCG/TCCACATTGAAGCCGGAGAC	77
ND2	ENSOCUG00000029090	AGGAATAGCCCCCTTCCACT/CCACCTCAACCGCCAACTAT	189
COX2	ENSOCUG00000029099	TCCGCATGCTAATCTCCTCG/TCCGGGAATGGCATCTGTTT	83
COX1	ENSOCUG00000029096	TACCCCGACGGTACTCAGAC/TTGAGGCGAAGGCTTCTCAG	129
EBF1	ENSOCUG00000008985	ACATGCGGAGATTCCAGGTC/TCCAGATAAGAGGGCGTACCT	154
PPARG	ENSOCUG00000013194	GAGGACATCCAGGACAACC/GTCCGTCTCCGTCTTCTTT	168
CEBPB	ENSOCUG00000024667	CACAGTGACGAGTACAAGATCCG/\GACAGCTGCTCCACCTTCTTCT	155
RXRG	ENSOCUG00000005512	ACTGCCTCATTGACAAGCGT/CTCCACGGGCATGTCTTCAT	173
BCL6	ENSOCUG00000005429	CCCATGCCTGCTGAGTACAA/GTCATTCCGAGCCTCCTCTG	114
RN18S	ENSOCUG00000027166	ATCAGATACCGTCGTAGTTC/TTCCGTCAATTCCTTTAAG	167
