# Supplementary material for: Integrated Analysis of Transcriptome, microRNAs, and Chromatin Accessibility Revealed Potential Early B-Cell Factor1-Regulated Transcriptional Networks during the Early Development of Fetal Brown Adipose Tissues in Rabbits
Source: Cells. 2022 Aug 28;11(17):2675. doi: 10.3390/cells11172675 (PMC9454897; doi:10.3390/cells11172675)
Supplement: Supplementary file 1 [file cells-11-02675-s001.zip › cells-1832136-supplementary/Table S2.pdf]

Table S2 Summary of ATAC-seq, RNA-seq, and miRNA-seq data

Group	Sample	Library type	Clean reads	Clean reads Q20 (%)	Clean reads Q30 (%)	Total mapped reads (%)	Uniquely mapped reads (%)
G21	G21_1	ATAC-seq	97198126	98.50	95.01	94.81	63.71
G21	G21_2	ATAC-seq	95282534	98.52	95.22	95.16	63.75
G21	G21_3	ATAC-seq	127842454	98.54	95.10	95.11	63.91
G24	G24_1	ATAC-seq	127342618	97.60	93.56	89.07	59.56
G24	G24_2	ATAC-seq	140137806	97.97	94.38	93.55	66.08
G24	G24_3	ATAC-seq	109182412	97.41	93.18	93.42	63.54
G24	G24_4	ATAC-seq	148852286	97.95	94.31	94.72	66.76
G21	G21_1	RNA-seq	139978854	98.72	95.73	87.96	79.43
G21	G21_2	RNA-seq	140686996	98.72	95.73	88.66	80.01
G21	G21_3	RNA-seq	142858434	98.69	95.68	88.49	79.51
G24	G24_1	RNA-seq	97394516	98.68	95.65	88.01	77.37
G24	G24_2	RNA-seq	98715916	98.70	95.71	87.76	78.07
G24	G24_3	RNA-seq	90883292	98.69	95.67	87.99	77.72
G21	G21_1	miRNA-seq	8211208	100.00	100.00	-	-
G21	G21_2	miRNA-seq	6850736	100.00	100.00	-	-
G21	G21_3	miRNA-seq	8001523	100.00	100.00	-	-
G24	G24_1	miRNA-seq	5857131	100.00	100.00	-	-
G24	G24_2	miRNA-seq	6410357	100.00	100.00	-	-
G24	G24_3	miRNA-seq	6142669	100.00	100.00	-	-
