# Supplementary figures and images for: Integrated Analysis of Transcriptome, microRNAs, and Chromatin Accessibility Revealed Potential Early B-Cell Factor1-Regulated Transcriptional Networks during the Early Development of Fetal Brown Adipose Tissues in Rabbits
Source: Cells. 2022 Aug 28;11(17):2675. doi: 10.3390/cells11172675 (PMC9454897; doi:10.3390/cells11172675)

**A**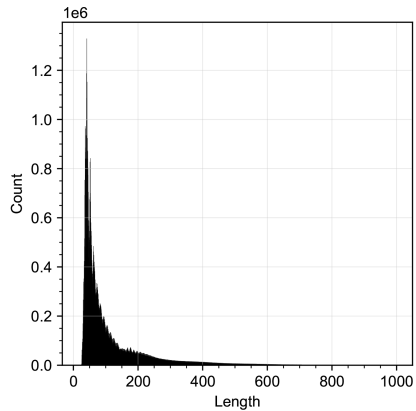**B**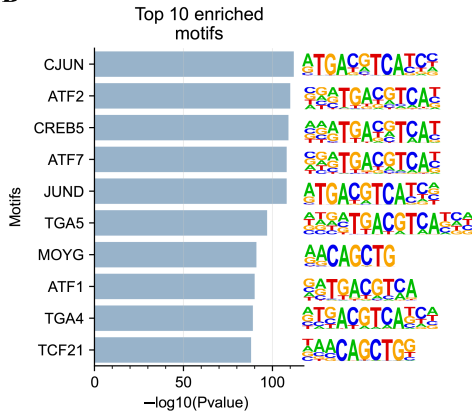

Supplement: Supplementary file 1 [file cells-11-02675-s001.zip › cells-1832136-supplementary/FigureS1-Length distribution of fragments in the ATAC-seq library and enrichment of TF binding motifs by all decreased peaks.pdf]

**A**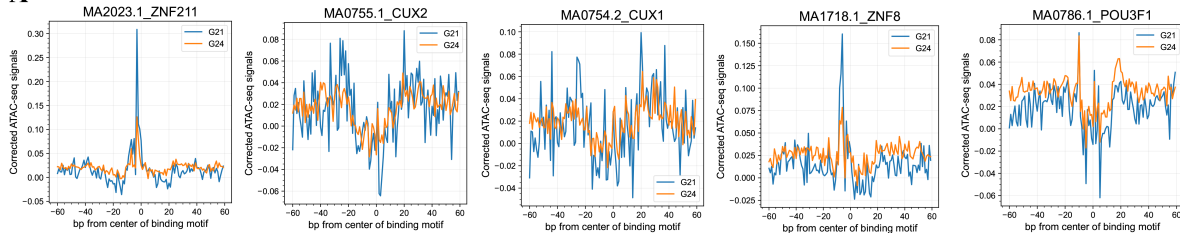**B**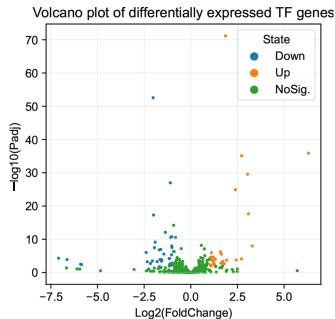**C**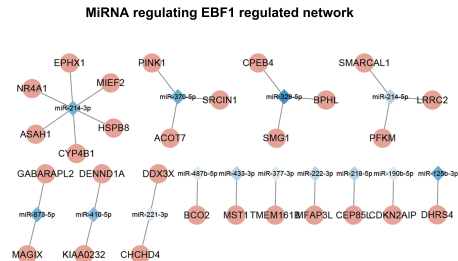**D**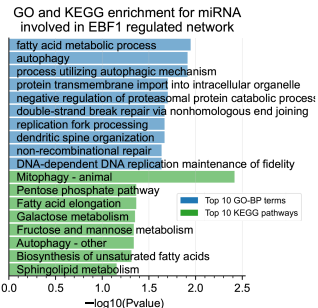

Supplement: Supplementary file 1 [file cells-11-02675-s001.zip › cells-1832136-supplementary/FigureS3-Genome-wide TF footprinting analysis during the early development of FBAT.pdf]

**A**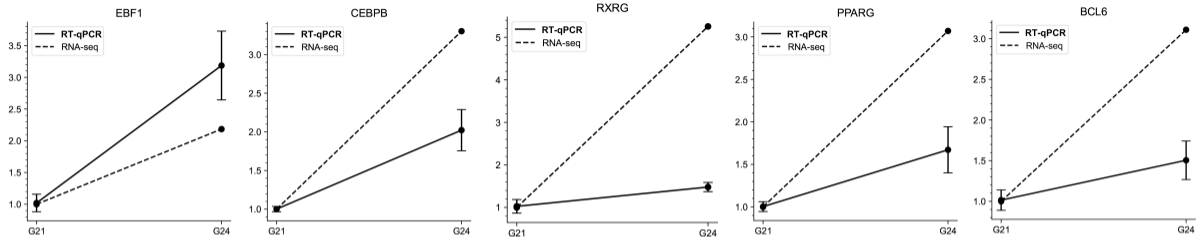**B**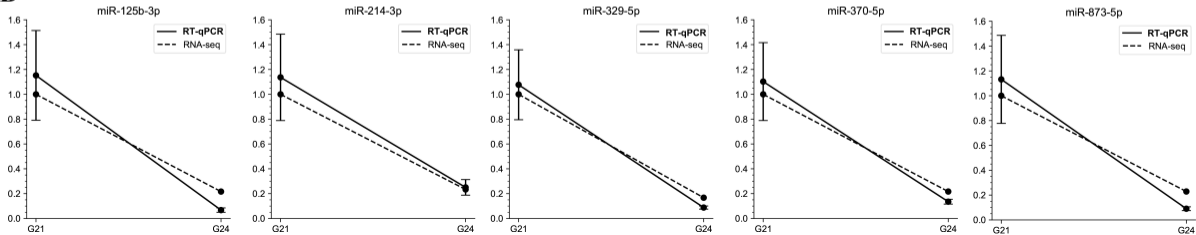

Supplement: Supplementary file 1 [file cells-11-02675-s001.zip › cells-1832136-supplementary/FigureS4-RT-qPCR validation of key TF genes and miRNAs involved in the EBF1- regulated gene net.pdf]
